# Prevalence of extended-spectrum beta-lactamase genes in Acinetobacter baumannii strains isolated from nosocomial infections in Tehran, Iran

**DOI:** 10.3205/dgkh000318

**Published:** 2019-01-25

**Authors:** Mojtaba Hanafi Abdar, Morovat Taheri-Kalani, Keyvan Taheri, Behzad Emadi, Amir Hasanzadeh, Abdolmajid Sedighi, Serve Pirouzi, Mansour Sedighi

**Affiliations:** 1 Department of Microbiology, Faculty of Medicine, Ilam University of Medical Sciences, Ilam, Iran; 2 Department of Microbiology, Science and Research Islamic Azad University, Damghan Branch, Damghan, Iran; 3 International Campus, Department of Microbiology, Faculty of Medicine, Iran University of Medical Sciences, Tehran, Iran; 4 Department of Microbiology, Faculty of Medicine, Maragheh University of Medical Sciences, Maragheh, Iran; 5 Department of Accounting, Faculty of Management and Accounting, Allameh Tabataba'i University, Tehran, Iran; 6 School of Hejab, Baneh management, Department of Kurdistan Education and Training, Department of Iran Education and training, Baneh, Iran; 7 Department of Microbiology, Faculty of Medicine, Iran University of Medical Sciences, Tehran, Iran; 8 Azarbaijan-Gharbi Regional Blood Transfusion Center, Urmia, Iran

**Keywords:** Acinetobacter baumannii, blaSHV, blaTEM, blaVEB, drug resistance, PCR

## Abstract

**Background and objectives:** *bla*_SHV_, *bla*_TEM_ and *bla*_VEB_ are a group of Extended-Spectrum Beta-Lactamase enzymes (ESBLs) which are able to hydrolyze Penicillins and some cephalosporin antibiotics. The present study evaluated the frequency of ESBL genes *bla*_SHV_, *bla*_TEM_ and *bla*_VEB_ in *Acinetobacter baumannii* strains isolated from nosocomial infections to outline the importance of these genes in antibiotic resistance.

**Methods:** One hundred *Acinetobacter baumannii* strains were isolated from different nosocomial infections. After antibiotic resistance evaluation with the Kirby-Bauer disc-diffusion method, the Minimum Inhibitory Concentration (MIC) of Ciprofloxacin was measured using the E-test method. Then, the ESBL producing strains were identified employing Combined Disk Methods. Finally, all isolates were evaluated with the Polymerase Chain Reaction (PCR) technique to detect the ESBL genes of interest.

**Results:** Out of 100 *Acinetobacter baumannii* isolates, 59% were ESBL positive according to the phenotypic method. The PCR assay could not detect the *bla*_SHV_ and *bla*_VEB_ genes in the studied isolates, but the presence of *bla*_TEM_ gene was demonstrated in 42% of the strains.

**Conclusion:** The high resistance to most antibiotics, the high prevalence of ESBLs-producing strains and also a high prevalence of *bla*_TEM_ gene in *A. baumannii* strains found in the current study gives cause for major concern about nosocomial infections in Iran because of the treatment complexity of these strains. Our results highlight the need for infection control measures to prevent the spread of resistant isolates, especially in hospitals.

## Zusammenfassung

**Hintergrund:** *bla*_SHV_, *bla*_TEM_ und *bla*_VEB_ gehören zur Gruppe der Extended-Spectrum Beta-Lactamase Enzyme (ESBLs) codierenden Gene, die Penicilline und einige Cephalosporine hydrolysieren können. In der vorliegenden Studie sollte die Häufigkeit dieser ESBL Gene bei *Acinetobacter baumannii* Stämmen, isoliert aus nosokomialen Infektionen, untersucht werden.

**Methode:** 100 *Acinetobacter baumannii* Stämme wurden bei verschiedenen nosokomialen Infektionen isoliert. Nach der Resistenzbestimmung gemäß der Agardiffusionsmethode nach Kirby Bauer wurde die Minimale Hemmkonzentration (MHK) von Ciprofloxacin mit der E-Test Methode bestimmt. Anschließend wurden die ESBL bildenden Stämme mit der Combination Disk-Methode identifiziert und abschließend die Gene mittels PCD detektiert.

**Ergebnisse:** 59% der Isolate waren phänotypisch ESVL positiv. Mittels PCR konnte nur das *bla*_TEM_ Gen detektiert werden.

**Schlussfolgerung:** Die Resistenz gegen die meisten Antibiotika, die hohe Prävalenz von ESBL Bildnern und des *bla*_TEM_ Gens bei Acinetobacter baumannii Stämmen ist besorgniserregend in Bezug auf die Behandlung nosokomialer Infektionen im Iran. Die Ergebnisse unterstreichen die Notwendigkeit von Infektionskontrollmaßnahmen zur Verhinderung der Ausbreitung resistenter Stämme vor allem in Krankenhäusern.

## Introduction

*Acinetobacter* species are aerobic gram-negative microorganisms responsible for various types of infections such as pneumonia, urinary tract infection and septicemia [1]. *Acinetobacter baumannii (A. baumannii)* is an important opportunistic pathogen that has caused global outbreaks of nosocomial infections [2].The Acenitobacter infections have been recognized as an emerging problem (one of major cause of nosocomial infections) and appeared to be associated with high mortality rates throughout the world [3].

The infections caused by *Acinetobacter* pathogens are often treated with Cephalosporins, including Ceftazidime, ceftriaxone, aminoglycosides such as tobramycin and amikacin, Carbapenems, and tetracycline. To date, however, most strains of *A. baumannii* have become increasingly resistant to almost all these currently available antibacterial agents due to the presence of mobile genetic elements, such as insertion sequences (ISs), plasmids, integrons and resistance islands [1], [2]. These mobile elements carry and transfer antimicrobial resistance genes to another bacterium and easily spread among various species and even genera of microorganisms. Furthermore, the transfer of these elements between chromosome and plasmid may facilitate the rapid spread of the resistance genes among different strains of bacteria. An increasing drug-resistance rate among *A. baumannii* strains is a major concern in hospitals worldwide [4], [5], [6]. The antimicrobial resistance in this nosocomial pathogen is mainly caused by beta-lactamase (β-lactamases) inactivating enzymes, alteration of membrane porin channels, and mutations that change cellular functions [5]. However, the most common mechanism of resistance is the production of hydrolytic enzymes of antimicrobial agents, including extended-spectrum beta-lactamases (ESBLs) that belong to Ambler classes A, D and B [7], [8], [9], [10]. ESBL-producing strains are mutant, acquired plasmid-mediated β-lactamases which demonstrate unique hydrolytic properties. They are enzymes with the potential to digest β-lactamase antibiotics, which possess Oxyimino groups such as Oxyimino-Cephalosporins. However, they are inhibited by Clavulanate and Tazobactam, β-lactamase inhibitors [4]. Clinical types of ESBL genes includes *bla*_SHV_, *bla*_TEM_, *bla*_VEB_, *bla*_KPC_, *bla*_PER_, *bla*_BEL-1_, *bla*_BES-1_, *bla*_SFO-1_, *bla*_TLA_ and *bla*_BIC_ that are associated with mobile genetic elements, predominantly plasmids [11]. The wide use of numerous new β-lactam antibiotics in recent decades has led to the emergence of ESBLs, which are mostly derivatives of TEM-1 (named for the patient, Temoneira) and SHV-1 (sulfhydryl variable) enzymes. They are capable of hydrolyzing a wide range of β-lactam antibiotics, except carbapenems that have been detected in *Acinetobacter* strains and many gram-negative bacteria [12].

Non-TEM-, non-SHV-derived ESBLs (VEB [Vietnamese extended-spectrum-beta-lactamase]) have been documented in *Acinetobacter* species from Europe and Asia but not yet from the Americas [11], [13]. The *bla*_VEB-1_ ESBL gene is belonging to class A β-lactamase and is located in a class 1 integron. These genetic structures are responsible for the expression of cassette-associated and mobile resistance genes, initially detected in *Enterobacteriaceae* and *Pseudomonas aeruginosa* from Southeast Asia [14], [15]. Subsequently, it has been described in clonally related *A. baumannii* isolates recovered during an outbreak that lasted 9 months (August 2001–April 2002) in the intensive care unit of a hospital in northern France. In these strains, the location of the *bla*_VEB-1_ gene on the chromosomes and integrons was identified [15]. This enzyme is associated with a high level of resistant to Cephems, Monobactams and Ceftazidime [16], [17]. In light of the above, the increasing rate of antibiotic consumption and its impact on treatment failure due to misuse or overuse of antibiotics by patients, and finally widespread drug-resistant strains particularly in hospitals, it is imperative to search for new means of controlling the increasing mortality rate based on the failure of drug therapy [18], [19], [20]. Regarding the growing importance of ESBLs in antibiotic resistance and its impact on treatment failure, this study was performed to evaluate the antimicrobial susceptibility patterns of clinical strains and determine the frequency of *bla*_SHV_, *bla*_TEM_ and *bla*_VEB_ genes in *A. baumannii* strains isolated from several hospitals in Tehran.

## Methods

### Bacterial strains

In this cross-sectional descriptive study, 100 isolated samples of *A. baumannii* were collected from 10 hospitals in Tehran (Imam Hoseyn, Mofid, Imam khomeyni, Shahid Labafi nazhad, Loghman, Khatamalanbya, Milad, Mostafa Khomeyni, Shariati and Motahari) from October 2014 to April 2015. The specimens were obtained from wounds, tracheae, blood, sputum, catheters, pleural fluid, urine and CSF of hospitalized patients. These isolates were collected from different hospital wards, including ICU, Burns, Internal Medicine, Emergency Medicine, Surgery, Medical, Neurological, BMT, CCU, Orthopedic and Maxillofacial Surgery wards. Replicated isolates from the same patients were excluded from the study. All isolates were identified morphologically and using conventional biochemical methods. The following phenotypic and bacteriological tests were used in this study: Gram staining, colony morphologies, McConkey’s agar, TSI, oxidase reaction, lysine decarboxylase, growth at 37°C and 45°C, hydrolyzed gelatin, citrate utilization, OF, and hemolysis on blood agar with 5% sheep blood. The obtained strains were preserved in tryptic soy broth (Merck, Darmstadt, Germany) containing 15% glycerol [21].

### Antibiotic susceptibility testing

Antibiotic susceptibility testing was performed using the agar diffusion test (Kirby-Bauer antibiotic testing), as recommended by the Clinical and Laboratory Standards Institute (CLSI), with disks containing Meropenem (MEM:10 *µ*g), Ceftazidime (CAZ: 30 *µ*g), Gatifloxacin (GAT: 10 *µ*g), Levofloxacin (LEV:10 *µ*g), Piperacillin/Tazobactam (P/T: 110 *µ*g), Ticarcillin-Clavulanate (T/C: 85 *µ*g), and Trimetoprim-Sulfametoxazol (TMS: 25 *µ*g) (MAST, Merseyside, U.K). *A. baumannii* ATCC19606 served as the control strain for antibiotic susceptibility testing [1], [22].

### Minimum inhibitory concentration (MIC)

The minimum inhibitory concentration (MIC) test was performed according to CLSI guidelines by the E-test method for Ciprofloxacin-resistant isolates on Mueller-Hinton agar medium. E-test-Imipenem strips were applied on the plates, and the plates were incubated at 37°C in air for 16 to 20 h [23]. Non-susceptible *A. baumannii* strains with MIC≥32 *µ*g/ml were considered as Ciprofloxacin resistant [24]. *A. baumannii* ATCC19606 and *Pseudomonas aeruginosa* ATCC27853 served as controls for the Ciprofloxacin E-test method.

### Phenotypic detection of ESBL-producing isolates

To detect ESBLs, all the isolates were tested employing the disk diffusion test (CDDT) containing Ceftazidime (CAZ) 30* µ*g and Cefotaxime (CTX) 30* µ*g with a combination of CAZ 30* µ*g+clavulanic acid (CA) 10* µ*g and CTX 30* µ*g+CA 10* µ*g per disc (Mast Group, Merseyside, UK). Zones of inhibition were compared with the CTX and CAZ discs alone and compared with the combined CAZ 30* µ*g+CA 10* µ*g and CTX 30* µ*g+CA 10* µ*g discs. An increase in zone diameter of ≥5 mm in the presence of clavulanic acid indicated the existence of ESBL in the test microorganism. *Escherichia coli* ATCC25922 and *Klebsiella pneumonia* ATCC700603 were used as negative and positive controls for ESBL production, respectively [25].

### DNA extraction

For molecular diagnosis, the total DNA of all *A. baumannii* isolates was extracted by the DNA extraction kit (Bioneer Company, Korea, Cat. number K-3032-2) specifically for the given bacterial colony.

### Detection of ESBL genes by PCR method

The genetic basis of the β-lactam resistance mediated by enzymatic mechanisms was investigated using the PCR method on all of the isolates phenotypically positive for ESBL. The PCR assay for detection of β-lactamase genes *bla*_SHV_, *bla*_TEM_ and *bla*_VEB_ was carried out using the previously reported specific oligonucleotide primers shown in Table 1 (Tab. 1). The PCR mixture contained the DNA template, Forward/Reverse primers, and master mix (Bioneer Co., Korea, Cat. number K-2016).

Amplification was carried out with the following thermocycling conditions. The cycling parameters used for the *bla*_SHV_ gene were: 94°C for 5 min, followed by 35 cycles of denaturation at 94°C for 30 sec, annealing at 56°C for 30 sec, extension at 72°C for 30 sec and a final extension at 72°C for 10 min. The parameters for the *bla*_TEM_ gene were: 94°C for 3 min, followed by 30 cycles of denaturation at 94°C for 1 min, annealing at 59°C for 1 min, extension at 72°C for 1 min and a final extension at 72°C for 10 min. The parameters used for the *bla*_VEB_ gene were: 94°C for 10 min, followed by 35 cycles of denaturation at 94°C for 1 min, annealing at 55°C for 1 min, extension at 72°C for 5 min and a final extension at 72°C for 10 min. The PCR product bands were analyzed electrophoretically on a 1% agarose gel at 100 V for 45–60 min in 1X TBE containing ethidium bromide. The results were examined under UV irradiation. The positive controls were three *A. baumannii* clinical strains containing *bla*_SHV_, *bla*_TEM_ and *bla*_VEB_ genes.

## Results

**Figure 1:**
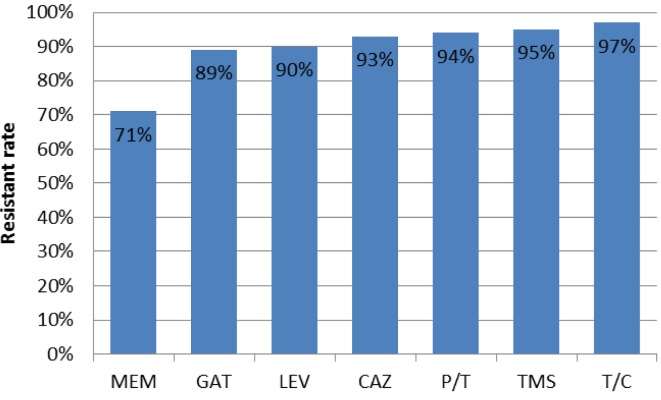
Antibiotic resistance pattern of *A. baumannii* isolates in this study

**Figure 2:**
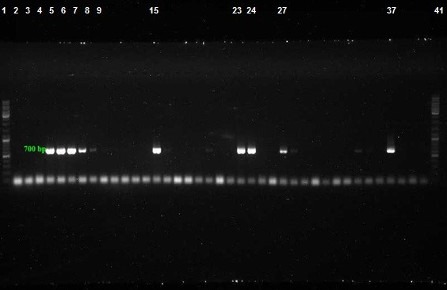
PCR assay for detection of *bla_TEM_* gene with product size: 700 bp. Lanes 2–36: Amplified products, Lane 37: Positive control, Lanes 1 and 40: DNA ladder 100 bp, Lane 38–40: Negative control

**Figure 3:**
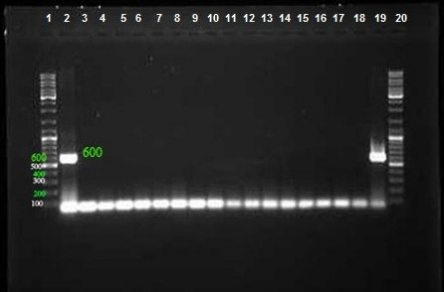
PCR assay for detection of *bla_VEB_* gene with product size: 600 bp. Lanes 3–17: Amplified products, Lanes 2 and 19: Positive control, Lanes 1 and 20: DNA ladder 100 bp, Lane 18: Negative control

**Figure 4:**
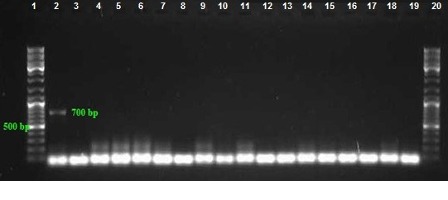
PCR assay for detection of *bla_SHV_* gene with product size: 700 bp. Lanes 3–18: Amplified products, Lane 2: Positive control, Lanes 1 and 20: DNA ladder 100 bp, Lane 19: Negative control

Among 100 collected strains, 57 and 43 were isolated from men and women, respectively. The majority and minority of isolates were obtained from wounds (40%) and CSF (3%), respectively (Table 2 (Tab. 2)). The highest isolation rate was associated with the ICU ward and the lowest with the orthopedic ward. As shown in Figure 1 (Fig. 1), sensitivity to Meropenem, Gatifloxacin and Levofloxacin was 29%, 11% and 10% respectively, which were most effective antibiotics against clinical isolates. The maximum rate of resistance was related to Ticarcilin-Clavelonic acid. The E-test illustrated that the MIC level of ciprofloxacin in all ciprofloxacin-resistant *A. baumannii isolates* (91%) was ≥32 µg/ml. In the phenotypic method, 59 (59%) isolates produced ESBL enzymes (increase of ≥7 mm in zone diameter of CAZ-clavulanic acid disks compared to CAZ disks. same value as for the CTX+clavulanic acid compared to Imipenem disks). In the molecular assay (PCR), *bla*_SHV_ and *bla*_VEB_ genes were not detected in any *A. baumannii* clinical isolates, but the presence of the *bla*_TEM_ gene was demonstrated in 42% of these strains (Figure 2 (Fig. 2), Figure 3 (Fig. 3) and Figure 4 (Fig. 4)).

## Discussion

*A. baumannii* is responsible for hospital-acquired infections and has recently become one of the most important healthcare-associated infections in hospitals. Infections caused by this pathogen often leads to significant mortality and morbidity [26]. Antimicrobial resistance in *A. baumannii* has become a worldwide problem, especially in healthcare centers and hospitalized patients. The emergence of clinical *A. baumannii* isolates with diverse antibiotic resistance phenotypes leads to difficulties in treating infections caused by this pathogen [3], [27]. In recent years, *A. baumannii* has been commonly reported as multiple-drug resistant (MDR); the resistance rates to Imipenem, Meropenem, Ceftazidime, Piperacillin/Tazobactam, Ciprofloxacin and Gentamicin in Latin America seem to be among the world's largest [28]. In the present study, resistance to Meropenem, Gatifloxacin, Levofloxacin, Ceftazidim, Pipracilin-Tazobactam, Cotrimoxazole and Ticarcilin-Clavelonic acid were 71%, 89%, 90%, 93%, 94%, 95% and 97%, respectively. In the study conducted by Fallah et al., the resistance of *A. baumannii* isolates against the tested antibiotics were as follows: 95.4% to Ceftazidime, 100% to Cefotaxime, 91.7% to Meropenem, 92.6% to ciprofloxacin, 95.4% to Piperacillin/Tazobactam and 98.1% to Cotrimoxazole [11]. According to a research by Al-Agamy et al., 100% of *A. baumannii* isolates were resistant to Cefepime, Cefotaxime and Ceftazidime, 70% were resistant to Imipenem and 85% of isolates were resistant to Ciprofloxacin [29]. In the study carried out by Chagas et al., Ciprofloxacin, Cefepime and Piperacillin/Tazobactam showed the highest resistance rate (99.4%), followed by Ceftazidime (97.4%), Imipenem (95.5%), Meropenem (94.2%) and Ampicillin/Sulbactam (93.5%), while Sulfamethoxazole/Trimethoprim showed the highest susceptibility rate (23.9%) [30]. Safari et al. reported that the resistance rates of *A. baumannii* isolates were 85%, 94%, 97%, 84%, 95%, and 98% against Imipenem, Meropenem, Ciprofloxacin, Amikacin, Piperacillin/Tazobactam and Cefotaxime, respectively [31]. The results of antimicrobial resistance in other studies are very close to those of the present work, demonstrating a high prevalence of *A. baumannii* resistance isolates and an increase in MDR strains in recent years.

Production of extended spectrum β-lactamases (ESBLs) is one of the most important resistance mechanisms of *A. baumannii* strains. A high prevalence of ESBL-enzyme-producing *A. baumannii* strains has been documented by various studies around the world [32], [33], [34], [35]. In our study, of 59 (59%) ESBL-positive *A. baumannii* strains tested by PCR method, the *bla*_TEM_ gene was detected in 42% of them, while *bla*_SHV_ and *bla*_VEB_ genes were not found in these isolates. In a study conducted by Fallah et al., 84.2% of the investigated *A. baumannii* strains were ESBL positive according to the combined disk diffusion test; the *bla*_VEB_ gene was found in 39.5% of these isolates [11]. In the study carried out by Pasterán et al. (2006) that investigated 21 ESBL-producing *A. baumannii* strains, the *bla*_VEB_ gene was presented in 10 (47.6%) isolates [13]. In a study conducted by Thapa et al., out of 37 *A. baumannii* clinical isolates, the *bla*_VEB_ was detected in 7 (9%) strains [14]. The disparate results between the current study and other research regarding the prevalence of the *bla*_VEB_ gene may be due to the small sample size in the present study and/or different geographical locations.

In the study by Chaudhary et al., of 250 *A. baumannii* isolates, 209 (83.6%) were ESBL producers. Among the ESBL genes, the prevalence of *bla*_TEM_ varied from 82% to 87%, followed by *bla*_SHV_ (67–78%) in all ESBL-producing isolates [1]. In contrast to our study, Chaudhary et al. showed a high prevalence of the *bla*_SHV_ gene, but the prevalence of the *bla*_TEM_ gene was similar to our results in terms of prevalence rate. In research conducted by AL-Thahab et al., 83.8% of *A. baumannii* isolates were positive in the ESBL test. Resistance to ampicillin among ESBL positive strains was 100%. Among these isolates, 25% and 8.3% harbored *bla*_SHV_ and *bla*_TEM_ genes, respectively, but the *bla*_VEB_ gene was not found in any of the strains [36]. These findings show that despite the high prevalence of ESBL-positive *A. baumannii* isolates, the spread of *bla*_SHV_ and *bla*_TEM_ genes in these isolates is very low. On the other hand, in the study by Asadollahi et al. on the 23 strains of *A. baumannii* isolated from burn-wound infections, the frequency of *bla*_TEM_ and *bla*_SHV_ genes was 43.4% and 4.3%, respectively [37]. The results of these studies closely resemble those of the present study. In the study by Ramoul et al., the presence of ESBLs in 23 *A. baumannii* strains was confirmed by the E-test. PCR assay detected *bla*_TEM_ in three strains, but *bla*_SHV_ was not observed in any of these isolates [22]. In a study by Al-Agamy et al., E-test strips for ESBLs were applied to 40 isolates, of which 30 (75%) strains yielded a positive result. The most prevalent ESBL gene was *bla*_TEM_, which was detected in 35 (87.5%) isolates; however, *bla*_SHV_ and *bla*_VEB_ genes were not found [29]. Moreover, in the study by Lopes et al., the PCR technique performed to detect beta-lactamase genes in 50 isolates showed that all isolates harbored the *bla*_TEM_ gene, while *bla*_SHV_ and *bla*_VEB_ genes were not found in any of the strains [38]. The results of these studies regarding the prevalence of *bla*T_EM_, *bla*T_EM_ and *bla*_VEB_ ESBL genes were similar to our results. This indicates that the prevalence of *bla*_TEM_ gene is higher than that of *bla*_VEB_ and *bla*_TEM_ genes in different regions. In a study by Koo et al., 35 MDR *A. baumannii* isolates were examined, and 7 antibiotic resistance gene determinants were investigated. They were unable to detect *bla*_TEM_, *bla*_SHV_ and *bla*_VEB_ beta-lactamase genes [39]. ESBL genes are commonly located on class 1 integrons and are mostly plasmid mediated, and thus transfer easily to other bacteria. This genetic transfer among bacteria causes more rapid spread of ESBL-producing strains. Therefore, the results of different studies can vary due to different regions in which the studies were performed, varied prevalence of pathogenic strains carrying resistance genes especially in hospitals, abusing/overusing of antimicrobial drugs by patients, and study design in term of methodology and sample size [5].

## Conclusions

The results of this study reveal an alarming percentage of ESBL-producing strains occurring in Iran. ESBL-producing isolates have emerged as a major challenge, arising from overuse of expanded-spectrum Cephalosporins in hospitals and nursing homes. The high level of resistance to most antibiotics and high prevalence of *bla*_TEM_ genes in this study indicate the trend to increasing antibiotic resistance in our country and the complexity of treating infections caused by this organism. This study helps understand both the need for more caution in antibiotic consumption and the alarming rate of resistance. Future studies focus on investigation of other ESBL genes. It is necessary to seek a means of monitoring the ESBLs in healthcare settings to prevent the spread of resistant strains and facilitate the selection of appropriate antibiotics for patient treatment.

## Abbreviations

- ESBLs: Extended-Spectrum Beta-Lactamase enzymes
- PCR: Polymerase Chain Reaction
- MDR: Multiple-drug resistant
- ISs: Insertion sequences
- TSI: Triple Sugar Iron
- OF: Oxidative-fermentative
- MEM: Meropenem
- CAZ: Ceftazidime
- GAT: Gatifloxacin
- LEV: Levofloxacin
- P/T: Piperacillin/Tazobactam
- T/C: Ticarcillin-clavulanate
- TMS: Trimetoprim-sulfametoxazol
- CLSI: Clinical and Laboratory Standards Institute
- MIC: Minimum Inhibitory Concentration
- CDDT: combination disk diffusion test
- TBE: Tris/Borate/EDTA

## Notes

### Acknowledgments

The authors extend their appreciation to the personnel of Iran and Shahed University of Medical Sciences for their cooperation and their technical assistance in this work.

### Competing interests

The authors declare that they have no competing interests.

## Figures and Tables

**Table 1:**
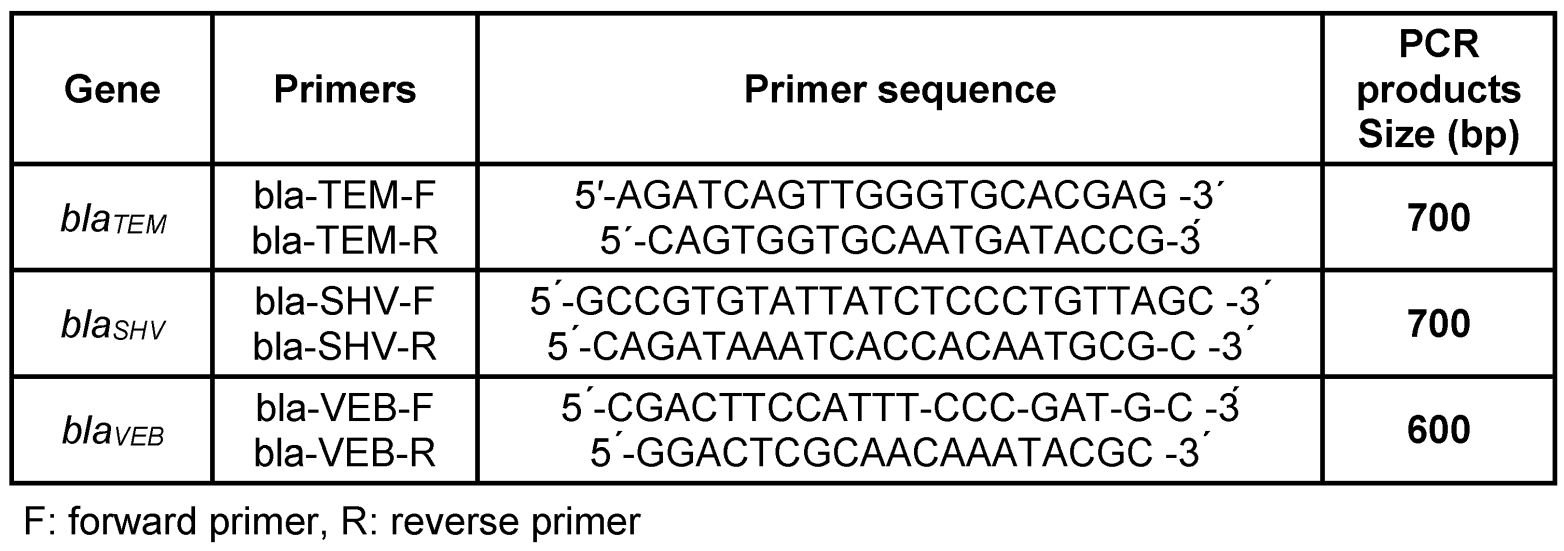
Oligonucleotides used in the study for each tested gene in PCR method

**Table 2:**
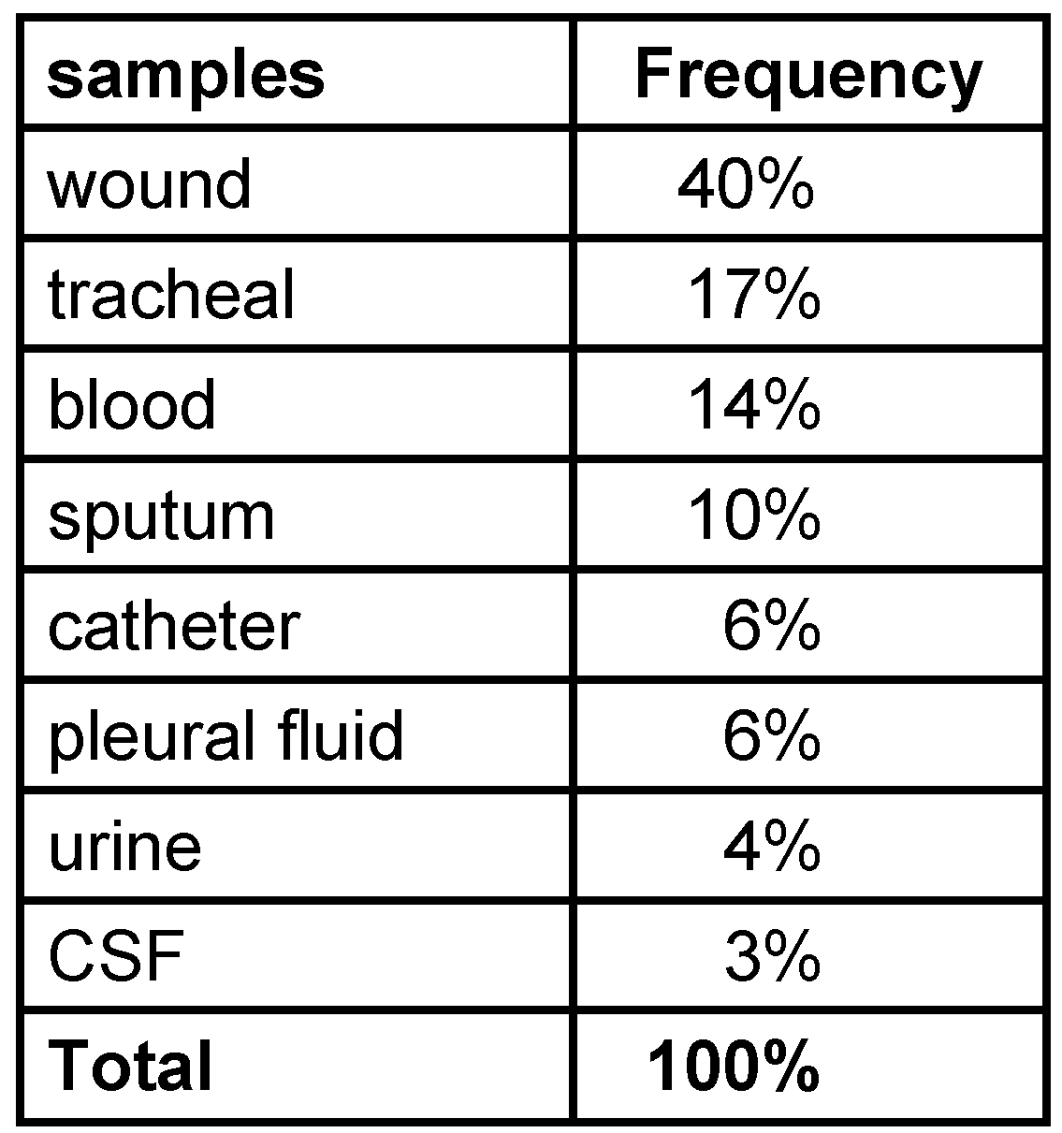
Frequency of A. baumannii isolates based on the type of samples
